# Towards a phenome-wide catalog of human clinical traits impacted by genetic ancestry

**DOI:** 10.1186/s13040-015-0068-y

**Published:** 2015-11-11

**Authors:** Logan Dumitrescu, Nicole A. Restrepo, Robert Goodloe, Jonathan Boston, Eric Farber-Eger, Sarah A. Pendergrass, William S. Bush, Dana C. Crawford

**Affiliations:** 1 Center for Human Genetics Research, Department of Molecular Physiology and Biophysics, Vanderbilt University, Nashville, TN 37232 USA; 2 Center for Systems Genomics, Department of Biochemistry and Molecular Biology, The Pennsylvania State University, University Park, PA 16802 USA; 3 Institute for Computational Biology, Department of Epidemiology and Biostatistics, Case Western Reserve University, Wolstein Research Building, 2103 Cornell Road, Suite 2527, Cleveland, OH 44106 USA

## Abstract

**Background:**

Racial/ethnic differences for commonly measured clinical variables are well documented, and it has been postulated that population-specific genetic factors may play a role. The genetic heterogeneity of admixed populations, such as African Americans, provides a unique opportunity to identify genomic regions and variants associated with the clinical variability observed for diseases and traits across populations.

**Method:**

To begin a systematic search for these population-specific genomic regions at the phenome-wide scale, we determined the relationship between global genetic ancestry, specifically European and African ancestry, and clinical variables measured in a population of African Americans from BioVU, Vanderbilt University’s biorepository linked to de-identified electronic medical records (EMRs) as part of the Epidemiologic Architecture using Genomics and Epidemiology (EAGLE) study. Through billing (ICD-9) codes, procedure codes, labs, and clinical notes, 36 common clinical and laboratory variables were mined from the EMR, including body mass index (BMI), kidney traits, lipid levels, blood pressure, and electrocardiographic measurements. A total of 15,863 DNA samples from non-European Americans were genotyped on the Illumina Metabochip containing ~200,000 variants, of which 11,166 were from African Americans. Tests of association were performed to examine associations between global ancestry and the phenotype of interest.

**Results:**

Increased European ancestry, and conversely decreased African ancestry, was most strongly correlated with an increase in QRS duration, consistent with previous observations that African Americans tend to have shorter a QRS duration compared with European Americans. Despite known racial/ethnic disparities in blood pressure, European and African ancestry was neither associated with diastolic nor systolic blood pressure measurements.

**Conclusion:**

Collectively, these results suggest that this clinical population can be used to identify traits in which population differences may be due, in part, to population-specific genetics.

**Electronic supplementary material:**

The online version of this article (doi:10.1186/s13040-015-0068-y) contains supplementary material, which is available to authorized users.

## Introduction

Racial/ethnic differences for commonly measured clinical variables, such as cholesterol [1], body mass index [2], and hypertension [3], are well documented. Although the causes of these observed differences are unclear, it has been postulated that population-specific genetic factors may play a role [4]. The genetic heterogeneity of admixed populations such as African Americans provides a unique opportunity to identify genomic regions and variants associated with the clinical variability observed for diseases and traits across populations.

Previous studies have been primarily limited to genome-wide association studies (GWAS) stratified by race/ethnicity (self-reported and/or genetic ancestry) and admixture mapping studies of one or a handful of phenotypes in mostly epidemiologic collections. Both GWAS and admixture mapping studies offer the opportunity to identify population-specific and trans-population associations involving specific genetic variants or genomic regions. Other previous studies have directly tested for associations between race/ethnicity or genetic ancestry and specific phenotypes such as atrial fibrillation [5–7]. These latter studies offer the opportunity to identify and perhaps distinguish between genetic and cultural or environmental factors that may account for the differences in disease prevalence or incidence observed across populations. Despite the success of these studies, no study has begun a systematic search of associations between genetic ancestry and traits phenome-wide.

Large epidemiologic and clinical collections often contain hundreds to thousands of data points related to the health status of individuals. To begin a systematic search for these population-specific genomic regions at the phenome-wide scale, we as the Epidemiologic Architecture for Genes Linked to Environment (EAGLE) study determined the relationship between global genetic ancestry (percent European and African ancestry) and clinical variables measured in an African American population from BioVU, the Vanderbilt University biorepository linked to de-identified electronic medical records [8, 9]. We describe here the distribution of global European and African ancestry and significantly associated clinical traits among >11,000 African Americans from BioVU. Overall, these data suggest that systematic searches for relationships between genetic ancestry and disease outcomes and traits have the potential to prioritize phenotypes with evidence of strong population differences for further study.

## Methods

### Study population

The DNA samples and data described here are from Vanderbilt University’s BioVU, a biorepository linked to de-identified electronic medical records. The establishment of BioVU including the ethical and legal considerations has been described elsewhere [8, 10]. Briefly, BioVU is an opt-out clinical collection that includes DNA samples extracted from discarded blood drawn for routine care at Vanderbilt University Medical Center out-patient clinics. DNA samples are linked to a de-identified version of the patient’s electronic medical records known as the Synthetic Derivative. The Synthetic Derivative contains structured, semi-structured, and unstructured clinical data that can be used for research purposes. Race/ethnicity in BioVU is administratively assigned and has been previously shown to be highly concordant with genetic ancestry for European Americans and African Americans [11, 12].

### Genotyping

We as part of the EAGLE study accessed all DNA samples and data from non-European Americans within BioVU as of 2011 for genotyping. These data are collectively referred to here as “EAGLE BioVU” [9]. A total of 15,863 samples were targeted for Illumina Metabochip genotyping. The Illumina Metabochip is a 200,000 variant array designed for replicating genome-wide association study findings (index variants) and for fine mapping select GWAS findings for cardiovascular and metabolic traits and outcomes [13]. The EAGLE BioVU dataset was generated by the Vanderbilt DNA Resources Core, and genotype calls and quality control were performed by the Population Architecture using Genomic and Epidemiology (PAGE) Coordinating Center as previously described [9, 14].

### Phenotyping

We defined 36 phenotypes using a combination of billing (International Classification of Diseases 9 or ICD-9) codes, procedure (CPT) codes, labs, and clinical notes available in the Synthetic Derivative. All phenotypes described here were extracted for the genetic association studies as part of the larger PAGE I study [15] and will be available via dbGaP. Algorithms for body mass index [Goodloe R, Faber-Eger E, Boston J, Crawford DC, Bush WS: Reducing clinical noise for body mass index measures due to unit and transcription errors in the electronic medical record, in preparation]. electrocardiographic traits (QRS duration, PR interval, QT interval, QRS, and heart rate) [16], and type 2 diabetes [17] have been previously described. The other phenotypes were defined as follows:

#### Lipids

- Laboratory measurements were queried for high-density lipoprotein cholesterol (HDL-C), low density lipoprotein cholesterol (LDL-C), total cholesterol, and triglycerides.
- Records were also queried for calculated LDL-C.
- For each individual, median values were calculated for a) measurements taken when no medications are prescribed (“pre-medication” values) and b) measurements taken at first mention of medication and post mention of medication (“post-medication” values)
- Medication class and list: statins (also known as HMG CoA reductase inhibitors, atorvastatin (Lipitor®), fluvastatin (Lescol®), lovastatin (Mevacor®, Altoprev™), pravastatin (Pravachol®), rosuvastatin calcium (Crestor®), simvastatin (Zocor®), lovastatin + niacin (Advicor®), atorvastatin + amlodipine (Caduet®), and simvastatin + ezetimibe (Vytorin™); selective cholesterol absorption inhibitors (ezetimibe (Zetia®)); resins (cholestyramine (Questran®, Questran® Light, Prevalite®, Locholest®, Locholest® Light), colestipol (Colestid®), colesevelam Hcl (WelChol®)); fibrates (gemfibrozil (Lopid®), fenofibrate (Antara®, Lofibra®, Tricor®, and Triglide™), clofibrate (Atromid-S)); and niacin.

#### Kidney traits

- Laboratory measures were queried for albumin (UABM), serum albumin (ALB), urinary albumin (UAlb, AlbCnc), albumin/creatinine ratio (AlbCre, SUA/C), creatinine from blood (Creat), creatinine from urine (URCRE, SUCrea, Creat1), urea nitrogen blood (BUN), and uric acid.
- For each individual, median values were calculated if more than one value was available in the Synthetic Derviative.

#### Primary essential hypertension

- Case definition 1: Individuals on hypertensive medication (list under systolic and diastolic blood pressure) ***and*** ICD-9 codes 401.* ***or*** mention of “high blood pressure” or “hypertension” in problem list
- Case definition 2: individuals not on hypertensive medication (list under systolic and diastolic blood pressure) ***and*** >140/90 systolic/diastolic blood pressure readings
- “Baseline” hypertension was defined as an individual’s hypertension status at first clinical visit.
- “Lifetime” hypertension status was defined as an individual’s hypertension status over the course of the clinical records available in the Synthetic Derivative.

#### Systolic and diastolic blood pressure

- Measurements for pregnant individuals were excluded.
- In-patient measurements were excluded.
- For each individual, “baseline” blood pressure measures represent the first systolic and diastolic blood pressure mentioned in the Synthetic Derivative.
- For each individual, median values were calculated for a) measurements taken when no medications are prescribed (“pre-medication” values) and b) measurements taken at first mention of medication and post mention of medication (“post-medication” values).
- Medications: angiotensin converting enzyme inhibitors, angiotensin receptor blockers, beta blockers, non-dihydropyridine calcium channel blockers, dihydropyridine calcium channel blockers, hydralazine, minoxidil, central alpha agonists, direct renin antagonists, aldosterone antagonists, alpha antagonists, diuretics (thiazides, K-sparing, and loop diuretics)
- Excluded medication: phentolamine, phenoxybenzamine

#### Type 2 diabetes related traits

- Laboratory measures were queried for glucose, glycated hemoglobin, and insulin.
- For each individual, median values were calculated if more than one value was available in the Synthetic Derivative.

### Statistical methods

EAGLE BioVU Metabochip genotype data as part of the PAGE I study were first subjected to quality control, including the identification of genetic ancestry outliers via EIGENSTRAT, through the PAGE I Coordinating Center [14]. We selected 7,655 uncorrelated SNPs from among the ancestry informative SNPs described by the PAGE I Coordinating Center [14] assayed by the Illumina Metabochip to estimate global genetic ancestry among administratively assigned African Americans in quality-controlled EAGLE BioVU genotype data using STRUCTURE v2.3.4 (K = 3) [18]. Data from 395 International HapMap samples represented CEU, YRI, and CHB/JPN were downloaded from the International HapMap Project and included in STRUCTURE runs as predefined parental clusters. Percent European ancestry was determined by STRUCTURE for each individual and used as the independent variable in tests of association.

Tests of association were performed using logistic or linear regression for 36 outcomes or traits where global ancestry was the independent variable. Primary essential hypertension (baseline and lifetime) and type 2 diabetes were binary outcomes; all other outcomes were continuous. Similar to the high-throughput PheWAS pipeline proposed by the PAGE I study [19], analyses were not adjusted for covariates. Also similar to the PAGE I study PheWAS pipeline [19], all continuous traits were tested for an association as untransformed and transformed (1+ natural logarithm) variables. Analyses were performed using SAS version 9.2 (Cary, NC) and R (version 3.2.0) in R Studio (version 0.99.441).

We estimated local ancestry using LAMP [20] for three fine-mapped regions on the Metabochip representing the three strongest associations with global European ancestry ranked by p-values from the tests of association: QRS duration, QT interval, and BMI. For these three traits, we identified a GWAS-significant variant in the NHGRI GWAS Catalog [21] that was assayed by the Metabochip and further fine-mapped by the Metabochip. From this search, we identified *SCN10A* rs6801957 (QRS duration) [22], *NOS1AP* rs12143842 (QT interval) [23–27], and *FTO* rs1558902 (BMI) [28]. We then considered assayed genetic variants within a 50 kb window of each of these GWAS-index variants (*SCN10A* chr3:38664112–38860101; *NOS1AP* chr1:160256929–160654852; *FTO* chr16:52245615–52755879) for local ancestry estimation using LAMP assuming 10 generations with an alpha at 0.2 and 0.8 and with a recombination rate of 1.0x10^−9^. We tested for associations between percent local European and African ancestry and each of the three traits untransformed and transformed (1+ natural logarithm).

## Results

EAGLE BioVU characteristics are given in Table 1. On average, most are female and relatively young. The largest non-European descent group is African American (*n* = 11,166), consistent with Davidson County, Tennessee population characteristics based on the 2010 US Census. The average individual in EAGLE BioVU had approximately 82 clinic visits and 147 ICD-9 codes available in his/her medical record.

**Table 1:** EAGLE BioVU characteristics (*n* = 15,863)

% female	63.35
Mean (±SD) age in years	37 ± 20.46
African American	73.06 %
Hispanic	10.87 %
Asian	7.12 %
Other race/ethnicity	8.95 %
Mean (range) clinical visits	81.8 (1 – 1,456)
Mean (range) ICD-9 codes	147.3 (1 – 3,617)

Demographic and summary clinical characteristics are given for the study population

*Abbreviations*: standard deviation (*SD*), international classification of diseases (*ICD-9*)

**Fig. 1:**
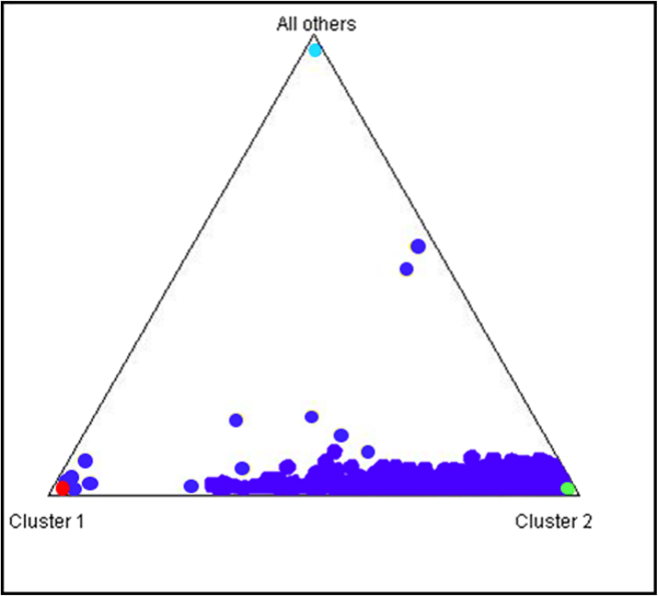
Genetic ancestry and EAGLE BioVU African Americans. Global genetic ancestry was estimated in 11,166 African Americans in EAGLE BioVU using 7,655 SNPs from the Illumina Metabochip and STRUCTURE assuming K = 3. The STRUCTURE plot is anchored by 395 International HapMap Project samples (CEU in red, YRI in green, and CHB/JPT in light blue)

**Fig. 2:**
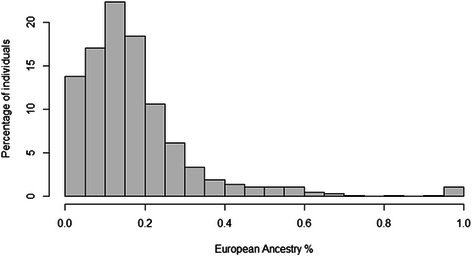
Distribution of percent European global ancestry among. African Americans in EAGLE BioVU. STRUCTURE was used to estimate global genetic ancestry using 7,655 SNPs assayed on the Illumina Metabochip and assuming K = 3. Plotted are the global European ancestry estimates for 11,166 African Americans in EAGLE BioVU where the x-axis represents the % global European ancestry and the y-axis represents the % of the total African American sample in EAGLE BioVU

**Fig. 3:**
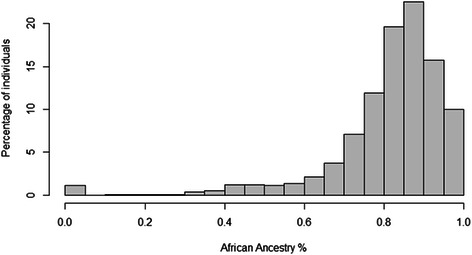
Distribution of percent African global ancestry among. African Americans in EAGLE BioVU. STRUCTURE was used to estimate global genetic ancestry using 7,655 SNPs assayed on the Illumina Metabochip and assuming K = 3. Plotted are the global West African ancestry estimates for 11,166 African Americans in EAGLE BioVU where the x-axis represents the % global African ancestry and the y-axis represents the % of the total African American sample in EAGLE BioVU

For each African American in EAGLE BioVU, we estimated percent European global genetic ancestry using 7,655 SNPs from the Metabochip and STRUCTURE. As expected, the African American population in EAGLE BioVU represents a complex, two-way admixture event of European and African-descent populations (Figs. 1, 2 and 3). Percent West African ancestry ranged from 0 % to 100 % with a mean of 81 % and interquartile range of 12.7 % (Fig. 3). Percent European ancestry ranged from 0 % to 100 % with a mean of 17.2 % and interquartile range of 12.8 %. The average percent European ancestry estimated here is consistent with other estimates for African Americans [5, 7].

Given that race/ethnicity is administratively assigned and not self-reported [11, 12], it is possible that individuals at the extremes of the global genetic ancestry distribution have been incorrectly assigned race/ethnicity. To explore this possibility, we extracted country of origin data from the clinical notes of EAGLE BioVU [Farber-Eger E, Goodloe R, Boston J, Bush WS, Crawford DC: Extracting country-of-origin from electronic medical records for gene-environment studies as part of the Epidemiologic Architecture for Genes Linked to Environment (EAGLE) study, in preparation]. Of the 1,159 individuals with less than 5 % European global genetic ancestry, only 44 had data on country of origin. Of these 44, 25 individuals with less than 5 % European global genetic ancestry have clinical notes with evidence that they are from African nations (Burundi, Ethiopia, Ghana, Kenya, Malawi, Nigeria, Rwanda, Senegal, Somalia, Sudan, Tanzania, and Uganda). Of the 114 individuals with >95 % European global genetic ancestry, only one individual had country of origin data (Haiti).

**Fig. 4:**
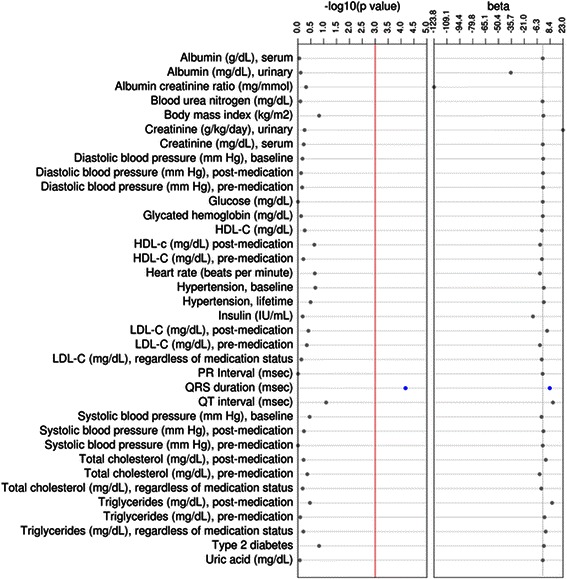
Continuous outcomes tested for an association with global European ancestry in African Americans from EAGLE BioVU. Tests of association were performed using linear regression between 33 continuous outcomes and percent. European global genetic ancestry among African Americans in EAGLE BioVU. Plotted are the –log_10_ of the p-value and the effect size (beta). Significant results are annotated in blue whereas non-significant results are annotated in gray. The red line represents the significance threshold at *p* = 0.001

We then performed sex-combined tests of association using logistic or linear regression for 36 outcomes or traits where European global ancestry was the independent variable (Table 2). Among the three binary outcomes considered, percent European ancestry was not associated with baseline hypertension (56 %; *p* = 0.21), lifetime hypertension (78 %; *p* = 0.39), or type 2 diabetes (12 %; *p* = 0.18). Among the remaining continuous outcomes considered, only QRS duration was significantly associated with percent European global genetic ancestry (*p* = 6.7x10^-5^, *n* = 837; Fig. 4). European ancestry was not associated with blood pressure measurements, kidney traits, type 2 diabetes associated measures, or any of the lipid traits (Table 2; Fig. 4). When all continuous traits were transformed, similar results were observed where only QRS duration was significantly associated with European global ancestry (*p* = 9.15x10^−5^; Additional file 1: Table S1).

**Table 2:** Outcomes tested for an association with global European ancestry in African Americans from EAGLE BioVU

Outcome (n)	% or mean (± SD)	OR or β (95 % CI or SE)	*P*-value
Hypertension, baseline (*n* = 6,422)	56 %	1.20 (0.90 – 1.59)	0.21
Hypertension, lifetime (*n* = 8,691)	78 %	1.23 (0.82 – 1.86)	0.32
Type 2 diabetes (*n* = 1,356)	12 %	1.32 (0.91 – 1.91)	0.15
Albumin (g/dL), serum (*n* = 8,094)	4.05 (0.51)	−0.001 (0.04)	0.88
Albumin creatinine ratio (mg/mmol) (*n* = 1,210)	14.0 (861.10)	−123.82 (176.60)	0.48
Albumin (mg/dL), urinary (*n* = 1,199)	16.7 (640.2)	−36.35(131.80)	0.78
Blood urea nitrogen (mg/dL) (*n* = 10,111)	11.00 (13.55)	−0.23 (0.93)	0.80
Body mass index (kg/m^2^) (*n* = 9,247)	27.25 (7.21)	0.74 (0.51)	0.15
Creatinine (mg/dL), serum (*n* = 10,288)	0.88 (1.99)	0.07 (0.13)	0.59
Creatinine (g/kg/day), urinary (*n* = 172)	70.00 (73.89)	23.05 (38.86)	0.55
Diastolic blood pressure (mm Hg), baseline (*n* = 10,025)	76.00 (15.68)	0.48 (1.07)	0.66
Diastolic blood pressure (mm Hg), post-medication (*n* = 5,653)	79.00 (10.06)	0.29 (0.90)	0.75
Diastolic blood pressure (mm Hg), pre-medication (*n* = 7,009)	75.00 (11.28)	0.38 (0.92)	0.68
Glucose (mg/dL) (*n* = 9,918)	96.00 (34.33)	0.04 (2.37)	0.99
Glycated hemoglobin (mg/dL) (*n* = 2,925)	6.30 (1.80)	0.07 (0.23)	0.75
HDL-C (mg/dL) (*n* = 5,096)	50.00 (16.99)	−1.02 (1.65)	0.54
HDL-c (mg/dL) post-medication (*n* = 2,097)	47.75 (16.56)	−2.95 (2.48)	0.23
HDL-C (mg/dL), pre-medication (*n* = 4,273)	51.00 (17.09)	−0.91 (1.81)	0.61
Heart rate (beats per minute) (*n* = 783)	76.00 (11.14)	−3.28 (2.67)	0.22
Insulin (IU/mL) (*n* = 343)	16.90 (74.36)	−11.15 (24.38)	0.65
LDL-C (mg/dL), regardless of medication status	99.00 (35.18)	−1.17 (3.42)	0.73
LDL-C (mg/dL), post-medication	96.00 (38.90)	4.95 (5.78)	0.39
LDL-C (mg/dL), pre-medication	105.00 (39.70)	−3.20 (4.26)	0.45
PR Interval (msec) (*n* = 781)	159.00 (17.92)	−0.12 (4.30)	0.98
***QRS duration (msec) (n = 837)***	***82.00 (8.65)***	***7.98 (1.99)***	***6.7x10*** ^-5^
QT interval (msec) (*n* = 783)	376.00 (27.84)	11.62 (6.66)	0.08
Systolic blood pressure (mm Hg), baseline (*n* = 10,025)	125.00 (22.54)	−1.43 (1.54)	0.35
Systolic blood pressure (mm Hg), post-medication (*n* = 5,653)	132.00 (16.03)	0.78 (1.43)	0.58
Systolic blood pressure (mm Hg), pre-medication (*n* = 7,009)	123.00 (16.38)	−0.0003 (1.34)	0.99
Total cholesterol (mg/dL), regardless of medication status (*n* = 5,439)	175.00 (39.48)	−1.65 (3.70)	0.65
Total cholesterol (mg/dL), post-medication (*n* = 2,150)	173.50 (44.22)	3.48 (6.58)	0.60
Total cholesterol (mg/dL), pre-medication (*n* = 4,642)	181.00 (44.86)	−3.57 (4.55)	0.43
Triglycerides (mg/dL), regardless of medication status (*n* = 5,269)	98.00 (69.46)	3.34 (6.61)	0.61
Triglycerides (mg/dL), post-medication (*n* = 2,115)	110.00 (74.07)	10.63 (11.07)	0.34
Triglycerides (mg/dL), pre-medication (*n* = 4,445)	97.00 (79.25)	2.00 (8.20)	0.81
Uric acid (mg/dL) (*n* = 2,465)	5.40 (2.18)	0.06 (0.29)	0.84

Tests of association were performed using logistic and linear regression between 36 outcomes and percent European global genetic ancestry among African Americans in EAGLE BioVU (*n* = 11,166). Descriptive statistics as well as summary statistics of all associations are shown for each outcome tested.

*Abbreviations*: odds ratio (*OR*), standard deviation (*SD*), standard error (*SE*)

Significant associations are bolded and italicized

**Fig. 5:**
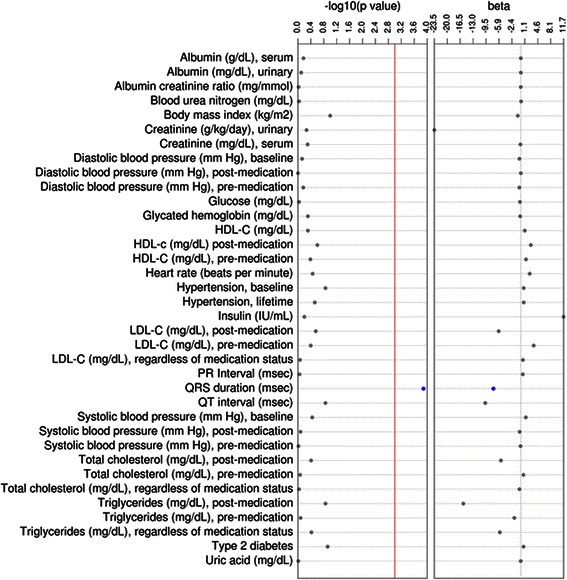
Continuous outcomes tested for an association with global African ancestry in African Americans from EAGLE BioVU. Tests of association were performed using linear regression between 33 continuous outcomes and percent. African global genetic ancestry among African Americans in EAGLE BioVU. Plotted are the –log_10_ of the p-value and the effect size (beta). Significant results are annotated in blue whereas non-significant results are annotated in gray. The red line represents the significance threshold at *p* = 0.001

We also performed sex-combined tests of association using logistic or linear regression for the same 36 outcomes or traits where African global ancestry was the independent variable (Table 3; Additional file 1: Table S2). Like the European global ancestry analyses (Table 2), the only significant association identified was for QRS duration (*p* = 1.3x10^−4^; Fig. 5). The genetic effect for the association identified between African global ancestry and QRS duration (β = −7.45; standard error 1.93) was similar in magnitude but opposite in direction compared with the association identified for European global ancestry (β = 7.98; standard error = 1.99; Table 2; Fig. 4).

**Table 3:** Outcomes tested for an association with global African ancestry in African Americans from EAGLE BioVU

Outcome (n)	% or median (± SD)	OR or β (95 % CI or SE)	*P*-value
Hypertension, baseline (*n* = 6,422)	56 %	0.81 (0.61 – 1.07)	0.14
Hypertension, lifetime (*n* = 8,691)	78 %	0.81 (0.54 – 1.21)	0.30
Type 2 diabetes (*n* = 1,356)	12 %	0.75 (0.52 – 1.08)	0.12
Albumin (g/dL), serum (*n* = 8,094)	4.05 (0.51)	0.02 (0.04)	0.67
Albumin creatinine ratio (mg/mmol) (*n* = 1,210)	14.00 (861.08)	0.01 (0.17)	0.95
Albumin (mg/dL), urinary (*n* = 1,199)	16.7 (640.2)	0.04 (0.16)	0.79
Blood urea nitrogen (mg/dL) (*n* = 10,111)	11.00 (13.55)	0.10 (0.90)	0.92
Body mass index (kg/m^2^) (*n* = 9,247)	27.25 (7.21)	−0.81 (0.50)	0.10
Creatinine (mg/dL), serum (*n* = 10,288)	0.88 (1.99)	−0.09 (0.13)	0.50
Creatinine (g/kg/day), urinary (*n* = 172)	70.00 (73.89)	−23.54 (38.74)	0.54
Diastolic blood pressure (mm Hg), baseline (*n* = 10,025)	76.00 (15.68)	−0.35 (1.05)	0.74
Diastolic blood pressure (mm Hg), post-medication (*n* = 5,653)	79.00 (10.06)	0.01 (0.88)	0.99
Diastolic blood pressure (mm Hg), pre-medication (*n* = 7,009)	75.00 (11.28)	−0.37 (0.90)	0.68
Glucose (mg/dL) (*n* = 9,918)	96.00 (34.33)	−0.22 (2.30)	0.92
Glycated hemoglobin (mg/dL) (*n* = 2,925)	6.30 (1.80)	−0.15 (0.22)	0.49
HDL-C (mg/dL) (*n* = 5,096)	50.00 (16.99)	1.09 (1.60)	0.49
HDL-c (mg/dL) post-medication (*n* = 2,097)	47.75 (16.55)	2.74 (2.38)	0.25
HDL-C (mg/dL), pre-medication (*n* = 4,273)	51.00 (17.09)	1.43 (1.73)	0.41
Heart rate (beats per minute) (*n* = 783)	76.00 (11.14)	2.44 (2.58)	0.35
Insulin (IU/mL) (*n* = 343)	16.90 (74.36)	11.67 (24.25)	0.63
LDL-C (mg/dL), regardless of medication status	99.00 (35.18)	0.58 (3.31)	0.86
LDL-C (mg/dL), post-medication	96.00 (38.90)	−6.00 (5.55)	0.28
LDL-C (mg/dL), pre-medication	105.00 (39.70)	3.52 (4.11)	0.40
PR Interval (msec) (*n* = 781)	159.00 (17.92)	0.56 (4.16)	0.89
***QRS duration (msec) (n = 837)***	***82.00 (8.65)***	***−7.45 (1.93)***	***1.3x10-4***
QT interval (msec) (*n* = 783)	376.00 (27.84)	−9.66 (6.45)	0.14
Systolic blood pressure (mm Hg), baseline (*n* = 10,025)	125.00 (22.54)	1.37 (1.50)	0.36
Systolic blood pressure (mm Hg), post-medication (*n* = 5,653)	132.00 (16.03)	−0.30 (1.40)	0.83
Systolic blood pressure (mm Hg), pre-medication (*n* = 7,009)	123.00 (16.38)	−0.06 (1.31)	0.96
Total cholesterol (mg/dL), regardless of medication status (*n* = 5,439)	175.00 (39.48)	−0.35 (3.57)	0.92
Total cholesterol (mg/dL), post-medication (*n* = 2,150)	173.50 (44.22)	−5.40 (6.31)	0.39
Total cholesterol (mg/dL), pre-medication (*n* = 4,642)	181.00 (44.86)	0.73 (4.38)	0.86
Triglycerides (mg/dL), regardless of medication status (*n* = 5,269)	98.00 (69.46)	−5.70 (6.40)	0.38
Triglycerides (mg/dL), post-medication (*n* = 2,115)	110.00 (74.07)	−15.64 (10.62)	0.14
Triglycerides (mg/dL), pre-medication (*n* = 4,445)	97.00 (79.25)	−1.75 (7.93)	0.83
Uric acid (mg/dL) (*n* = 2,465)	5.40 (2.18)	−0.01 (0.28)	0.97

Tests of association were performed using logistic and linear regression between 36 outcomes and percent African global genetic ancestry among African Americans in EAGLE BioVU (*n* = 11,166). Descriptive statistics as well as summary statistics of all associations are shown for each outcome tested.

*Abbreviations* odds ratio (*OR*), standard deviation (*SD*), standard error (*SE*)

Significant associations are bolded and italicized

For the three most significant test of associations between global ancestry and the outcomes and traits considered here (QRS duration, QT interval, and BMI; Table 2), we estimated local ancestry using LAMP [20] in the three fine-mapped regions (*SCN10A*, *NOS1AP*, and *FTO*) and tested for associations with local European and African ancestry. In this sample of African Americans, the QT interval associated *NOS1AP* region had on average the least European ancestry (16.7 %) compared with the BMI associated *FTO* region (23.6 %) and the QRS duration associated *SCN10A* region (24.6 %). None of the tests of association between local ancestry and the three traits was significant at *p* < 0.05.

## Discussion

We extracted 36 health outcomes and traits from de-identified electronic medical records of 11,166 African Americans in EAGLE BioVU and tested each of these phenotypes for an association with percent European and percent African global genetic ancestry. In sex-combined analyses, we identified a significant association between QRS duration and both European and African ancestry albeit in opposite directions. Other than QRS duration, no other outcome or trait was associated with European or African ancestry in sex-combined analyses.

QRS duration is a cardiac conduction trait extracted from electrocardiograms. Normal QRS duration generally ranges from 70 to 100 milliseconds in most populations [16]. Abnormal or prolonged QRS duration (>120 msec) is used in the diagnosis of bundle branch block or ventricular rhythm. The significant association identified here with European and African ancestry is consistent with previous observations that African Americans tend to have a shorter QRS duration compared with European Americans [16]. The lack of association between global ancestry and QT interval, another cardiac conduction trait tested here, is consistent with a similar study in African Americans from seven large population-based cohorts [25].

The present study had several limitations and strengths. A major limitation of this study is that global ancestry as opposed to local ancestry was estimated for each individual. The estimation of local ancestry will enable the identification of associations between phenotypes and specific genomic regions. However, estimation of local ancestry requires computational resources and dense genome-wide data. This dataset was limited to Metabochip data, which is dense only in fine-mapped regions and is sparse in non-fine mapped regions of the genome. Given the uneven genome-wide coverage, we were unable to confidently impute genome-wide data nor estimate local genetic ancestry at a genome-wide level for further study.

Despite these limitations, we did estimate local ancestry for three specific regions associated with three specific traits including QRS duration. In this sample of African Americans, QRS duration was not associated with local ancestry estimates for the *SCN10A* fine-mapped region. Variants in *SCN10A* have been associated with cardiac conduction (PR interval, QRS duration, and QT interval) in multiple populations including European-descent [22, 29–31] and Indian Asians [29]. For African Americans, variants in *SCN10A* have been strongly associated with PR interval [32]. Although a small GWAS in African Americans did not identify genome-wide significant associations between QRS duration and variants in *SCN10A*, the data suggest that the GWAS-index variants identified in European and Indian Asian populations generalize to African Americans with a similar genetic effect size and direction [33]. The lack of association between local genetic ancestry at *SCN10A* and QRS duration may be due to the fact that this locus is not responsible for the global ancestry signal detected here. This negative finding coupled with the lack of powerful GWAS in African Americans for QRS duration suggest that there may be other genetic variants and gene regions yet to be associated with this cardiac conduction trait in this population.

Another potential limitation for this global ancestry PheWAS is sample size and power. Overall, the sample size is moderate, with >11,000 African Americans with health-related data available for study. For individual phenotypes, however, sample size and power vary. The most powerful tests of association were limited to the laboratory values or vital signs routinely collected by the clinic, such as total cholesterol and blood pressure. However, even for these common measures, power may be adversely impacted by imprecise phenotype efforts when extracting data from electronic medical records. For example, it is assumed but not known if laboratory measures such as the lipid traits or type 2 diabetes traits were taken while the individual was fasting. Likewise, blood pressure measurements can be impacted by prescription medication noncompliance and diurnal effects. While care is taken to account for these factors in the phenotyping process, it is likely that all phenotypes extracted from electronic medical records have some degree of unintended phenotypic heterogeneity that will impact statistical power.

A major strength of this study is that it is one of the few large, clinical collections available for admixed populations such as African Americans. BioVU continues to accrue clinical data linked to DNA samples already collected as well as clinical data for new DNA samples representing new patients entering the Vanderbilt University Medical Center system. The accrual of these new data, samples, and eventually genetic data make this a potentially powerful dataset for further research in genetics and health disparities. Another major strength of the current study is electronic phenotyping using structured and unstructured data available in the EMR. Most previous PheWAS in clinical populations with available EMR data have been limited to billing (ICD-9) codes [30, 34–36]. Here, we take full advantage of the richness of the EMR to define cases and controls for select binary traits as well as to extract laboratory values for consideration as outcome variables. The specific variables for phenotyping and analysis in this PheWAS were chosen based on anticipated downstream studies in PAGE I [15]. While 36 phenotypes represent only a fraction of the phenome compared with a PheWAS based on ICD-9 codes, many of the phenotypes considered here have not yet been explored in published PheWAS. Also, limiting this PheWAS to 36 phenotypes reduces the number of statistical tests performed, which in turn lessens the impact of multiple testing when interpreting statistical significance of the results. The association observed between European global ancestry and QRS duration at *p* = 6.7x10^−5^ survives correction for multiple testing even if a conservative Bonferroni correction (*p* = 0.0014) is applied.

## Conclusions

We estimated global genetic ancestry in an admixed population and systematically searched for associations between European and African ancestry and clinical outcomes and traits mined from electronic medical records. We identified a significant association between European and African ancestry and QRS duration, and this association is supported by the known epidemiology of this electrocardiographic trait in diverse populations. Further general genomic discovery and study of local genetic ancestry are warranted to identify specific genomic regions associated with clinical outcomes in diverse populations.

## Additional file

Additional file 1: Table S1. Transformed continuous outcomes tested for an association with global European ancestry in African Americans from EAGLE BioVU. Tests of association were performed using linear regression between 33 transformed (1 + natural logarithm) continuous outcomes and percent European global genetic ancestry among African Americans in EAGLE BioVU (*n* = 11,166). Test results of all associations are shown for each outcome tested. Significant associations are bolded and italicized. Abbreviations: odds ratio (OR), standard deviation (SD), and standard error (SE). **Table S2.** Transformed continuous outcomes tested for an association with global African ancestry in African Americans from EAGLE BioVU. Tests of association were performed using linear regression between 33 transformed (1 + natural logarithm) continuous outcomes and percent African global genetic ancestry among African Americans in EAGLE BioVU (*n* = 11,166). Test results of all associations are shown for each outcome test. Significant associations are bolded and italicized. Abbreviations: odds ratio (OR), standard deviation (SD), and standard error (SE). (DOCX 23 kb)
