# Supplementary material for: Towards a phenome-wide catalog of human clinical traits impacted by genetic ancestry
Source: BioData Min. 2015 Nov 11;8:35. doi: 10.1186/s13040-015-0068-y (PMC4642611; doi:10.1186/s13040-015-0068-y)
Supplement: Additional file 1: Table S1. — Transformed continuous outcomes tested for an association with global European ancestry in African Americans from EAGLE BioVU. Tests of association were performed using linear regression between 33 transformed (1 + natural logarithm) continuous outcomes and percent European global genetic ancestry among African Americans in EAGLE BioVU (n = 11,166). Test results of all associations are shown for each outcome tested. Significant associations are bolded and italicized. Abbreviations: odds ratio (OR), standard deviation (SD), and standard error (SE). Table S2. Transformed continuous outcomes tested for an association with global African ancestry in African Americans from EAGLE BioVU. Tests of association were performed using linear regression between 33 transformed (1 + natural logarithm) continuous outcomes and percent African global genetic ancestry among African Americans in EAGLE BioVU (n = 11,166). Test results of all associations are shown for each outcome test. Significant associations are bolded and italicized. Abbreviations: odds ratio (OR), standard deviation (SD), and standard error (SE). (DOCX 23 kb) [file 13040_2015_68_MOESM1_ESM.docx]

**Supplementary Table 1. Transformed continuous outcomes tested for an association with global European ancestry in African Americans from EAGLE BioVU.**  Tests of association were performed using linear regression between 33 transformed (1 + natural logarithm) continuous outcomes and percent European global genetic ancestry among African Americans in EAGLE BioVU (n = 11,166). Test results of all associations are shown for each outcome test. Significant associations are bolded and italicized. Abbreviations: odds ratio (OR), standard deviation (SD), and standard error (SE).

| Outcome | Beta | SE | P-value |
| --- | --- | --- | --- |
| Albumin (g/dL), serum | -0.002 | 0.009 | 0.805 |
| Albumin creatinine ratio (mg/mmol) | -0.038 | 0.380 | 0.920 |
| Albumin (mg/dL), urinary | -0.156 | 0.362 | 0.667 |
| Blood urea nitrogen (mg/dL) | 0.004 | 0.040 | 0.912 |
| Body mass index (kg/m^2^) | 0.028 | 0.018 | 0.130 |
| Creatinine (mg/dL), serum | 0.021 | 0.027 | 0.435 |
| Creatinine (g/kg/day), urinary | -0.209 | 0.847 | 0.806 |
| Diastolic blood pressure (mm Hg), baseline | 0.003 | 0.020 | 0.897 |
| Diastolic blood pressure (mm Hg), post-medication | 0.003 | 0.012 | 0.795 |
| Diastolic blood pressure (mm Hg), pre-medication | 0.011 | 0.013 | 0.390 |
| Glucose (mg/dL) | 0.006 | 0.017 | 0.733 |
| Glycated hemoglobin (mg/dL) | 0.014 | 0.026 | 0.594 |
| HDL-C (mg/dL) | -0.013 | 0.031 | 0.676 |
| HDL-c (mg/dL) post-medication | -0.052 | 0.047 | 0.269 |
| HDL-C (mg/dL), pre-medication | -0.009 | 0.034 | 0.799 |
| Heart rate (beats per minute) | -0.043 | 0.035 | 0.227 |
| Insulin (IU/mL) | 0.002 | 0.285 | 0.996 |
| LDL-C (mg/dL), regardless of medication status | -0.008 | 0.046 | 0.869 |
| LDL-C (mg/dL), post-medication | 0.004 | 0.085 | 0.967 |
| LDL-C (mg/dL), pre-medication | -0.016 | 0.051 | 0.752 |
| PR Interval (msec) | 0.000 | 0.027 | 0.993 |
| *QRS duration (msec)* | ***0.093*** | ***0.024*** | ***9.15x10-5*** |
| QT interval (msec) | 0.030 | 0.018 | 0.091 |
| Systolic blood pressure (mm Hg), baseline | -0.020 | 0.022 | 0.356 |
| Systolic blood pressure (mm Hg), post-medication | 0.003 | 0.012 | 0.836 |
| Systolic blood pressure (mm Hg), pre-medication | 0.005 | 0.013 | 0.710 |
| Total cholesterol (mg/dL), regardless of medication status | -0.010 | 0.021 | 0.642 |
| Total cholesterol (mg/dL), post-medication | 0.021 | 0.036 | 0.560 |
| Total cholesterol (mg/dL), pre-medication | -0.020 | 0.024 | 0.397 |
| Triglycerides (mg/dL), regardless of medication status | 0.020 | 0.050 | 0.689 |
| Triglycerides (mg/dL), post-medication | 0.067 | 0.073 | 0.355 |
| Triglycerides (mg/dL), pre-medication | 0.011 | 0.057 | 0.845 |
| Uric acid (mg/dL) | 0.001 | 0.041 | 0.979 |

**Supplementary Table 2. Transformed continuous outcomes tested for an association with global African ancestry in African Americans from EAGLE BioVU.**  Tests of association were performed using linear regression between 33 transformed (1 + natural logarithm) continuous outcomes and percent African global genetic ancestry among African Americans in EAGLE BioVU (n = 11,166). Test results of all associations are shown for each outcome test. Significant associations are bolded and italicized. Abbreviations: odds ratio (OR), standard deviation (SD), and standard error (SE).

| Outcome | Beta | SE | P-value |
| --- | --- | --- | --- |
| Albumin (g/dL), serum | 0.005 | 0.008 | 0.577 |
| Albumin creatinine ratio (mg/mmol) | 0.024 | 0.364 | 0.948 |
| Albumin (mg/dL), urinary | 0.089 | 0.347 | 0.797 |
| Blood urea nitrogen (mg/dL) | -0.011 | 0.039 | 0.783 |
| Body mass index (kg/m^2^) | -0.031 | 0.018 | 0.083 |
| Creatinine (mg/dL), serum | -0.026 | 0.027 | 0.323 |
| Creatinine (g/kg/day), urinary | 0.141 | 0.845 | 0.868 |
| Diastolic blood pressure (mm Hg), baseline | 0.002 | 0.020 | 0.938 |
| Diastolic blood pressure (mm Hg), post-medication | 0.000 | 0.012 | 0.997 |
| Diastolic blood pressure (mm Hg), pre-medication | -0.010 | 0.012 | 0.430 |
| Glucose (mg/dL) | -0.006 | 0.016 | 0.691 |
| Glycated hemoglobin (mg/dL) | -0.022 | 0.025 | 0.382 |
| HDL-C (mg/dL) | 0.017 | 0.029 | 0.572 |
| HDL-c (mg/dL) post-medication | 0.053 | 0.045 | 0.245 |
| HDL-C (mg/dL), pre-medication | 0.033 | 0.032 | 0.311 |
| Heart rate (beats per minute) | 0.032 | 0.034 | 0.352 |
| Insulin (IU/mL) | -0.013 | 0.284 | 0.962 |
| LDL-C (mg/dL), regardless of medication status | -0.003 | 0.045 | 0.940 |
| LDL-C (mg/dL), post-medication | -0.028 | 0.082 | 0.732 |
| LDL-C (mg/dL), pre-medication | 0.014 | 0.049 | 0.781 |
| PR Interval (msec) | 0.002 | 0.026 | 0.933 |
| *QRS duration (msec)* | ***-0.088*** | ***0.023*** | ***1.6x10-4*** |
| QT interval (msec) | -0.025 | 0.017 | 0.148 |
| Systolic blood pressure (mm Hg), baseline | 0.023 | 0.021 | 0.279 |
| Systolic blood pressure (mm Hg), post-medication | 0.001 | 0.012 | 0.923 |
| Systolic blood pressure (mm Hg), pre-medication | -0.004 | 0.012 | 0.749 |
| Total cholesterol (mg/dL), regardless of medication status | -0.001 | 0.020 | 0.975 |
| Total cholesterol (mg/dL), post-medication | -0.032 | 0.035 | 0.352 |
| Total cholesterol (mg/dL), pre-medication | 0.008 | 0.023 | 0.713 |
| Triglycerides (mg/dL), regardless of medication status | -0.033 | 0.048 | 0.498 |
| Triglycerides (mg/dL), post-medication | -0.096 | 0.070 | 0.166 |
| Triglycerides (mg/dL), pre-medication | -0.011 | 0.056 | 0.850 |
| Uric acid (mg/dL) | 0.005 | 0.040 | 0.900 |
